# The Landscape of Nucleic-Acid-Based Aptamers for Treatment of Hematologic Malignancies: Challenges and Future Directions

**DOI:** 10.3390/bioengineering9110635

**Published:** 2022-11-02

**Authors:** Si Chun Wang, Xing Yi Yan, Chang Yang, Hua Naranmandura

**Affiliations:** 1 Department of Toxicology, School of Medicine and Public Health, Zhejiang University, Hangzhou 310058, China; 2 Department of Hematology, The First Affiliated Hospital, School of Medicine, Zhejiang University, Hangzhou 310058, China; 3 Liangzhu Laboratory, Zhejiang University Medical Center, Hangzhou 311121, China

**Keywords:** ApDCs, chemical linker, hematologic malignancy, target therapy

## Abstract

Hematologic malignancies, including leukemia, lymphoma, myeloproliferative disorder and plasma cell neoplasia, are genetically heterogeneous and characterized by an uncontrolled proliferation of their corresponding cell lineages in the bone marrow, peripheral blood, tissues or plasma. Although there are many types of therapeutic drugs (e.g., TKIs, chemotherapy drugs) available for treatment of different malignancies, the relapse, drug resistance and severe side effects due to the lack of selectivity seriously limit their clinical application. Currently, although antibody–drug conjugates have been well established as able to target and deliver highly potent chemotherapy agents into cancer cells for the reduction of damage to healthy cells and have achieved success in leukemia treatment, they still also have shortcomings such as high cost, high immunogenicity and low stability. Aptamers are ssDNA or RNA oligonucleotides that can also precisely deliver therapeutic agents into cancer cells through specifically recognizing the membrane protein on cancer cells, which is similar to the capabilities of monoclonal antibodies. Aptamers exhibit higher binding affinity, lower immunogenicity and higher thermal stability than antibodies. Therefore, in this review we comprehensively describe recent advances in the development of aptamer–drug conjugates (ApDCs) with cytotoxic payload through chemical linkers or direct incorporation, as well as further introduce the latest promising aptamers-based therapeutic strategies such as aptamer–T cell therapy and aptamer–PROTAC, clarifying their bright application, development direction and challenges in the treatment of hematologic malignancies.

## 1. Introduction

Hematologic malignancies are commonly classified into three main types: leukemia, lymphoma and myeloma [1]. Of note, leukemia is primarily bone marrow and peripheral-blood-based processes, whereas lymphomas are lymphatic system based and myeloma is predominantly bone-marrow-based diseases. Mechanistically, in hematopoietic progenitor cells, the genetic aberrations (i.e., point mutation, deletion or amplification of genetic material and gain, loss or translocation of chromosomal materials) frequently occur and are thought to be the main causes of hematologic malignancies [2,3,4]. These genetic aberrations can induce proto-oncogenes activation along with inactivation of tumor suppressor genes, which results in abnormal proliferation and self-renewal of hematopoietic progenitor cells, leading to an accumulation of immature blood cells in the bone marrow, tissues and peripheral blood [5]. 

Although, recently, many therapeutic options for hematologic malignancy treatment, such as tyrosine kinase inhibitors (TKIs) [6,7], chemotherapy and bone marrow transplantation, have significantly improved prognosis and survival of patients, some refractory (e.g., intrinsic resistance) and relapsed patients respond poorly to all current, available therapeutics [8,9,10]. Moreover, some potent cytotoxic chemotherapeutics can effectively kill cancer cells, but their severe side effects and systemic toxicity often limit their uses in broad terms due to lack of selectivity [11,12].

Several studies showed that targeted delivery of therapeutic agents into cancer cells through monoclonal antibodies (antibody–drug conjugates, ADCs) is considered as a promising strategy to tackle cancer and to increase therapeutic efficacy and reduce toxicity [13,14] because mAbs can recognize the biomarkers of a cancer cell and precisely deliver anticancer drugs into cells as drug carrier [15]. To date, more than ten ADCs have been approved for clinical applications, and about 80 ADCs are being evaluated in different phases of clinical trials [16]. Mylotarg (gemtuzumab ozogamicin), a CD33-targeted monoclonal antibody conjugated with cytotoxic drug calicheamicin, was approved for CD33-positive acute myeloid leukemia (AML) treatment. It represents a successful achievement for the site-specific delivery of cytotoxic agents into target leukemia cells through antibody-antigen recognition [17]. Beyond doubt, monoclonal antibodies (mAbs) have many advantages as a targeted molecule for cancer treatment, but they also have some shortcomings such as low stability owing to the protein natural properties, high immunogenicity, high cost and others [18,19,20]. Thus, novel, targeted drug delivery systems urgently need to be explored to overcome these disadvantages.

On the other hand, nucleic-acid-based drugs such as antisense oligonucleotides and aptamers are emerging as potential therapeutics for different diseases including leukemia [21,22]. Among them, aptamers, a special class of single-stranded DNA or RNA oligonucleotides discovered in nature as well as in laboratory, are beginning to be investigated for clinical use [23]. Similar to monoclonal antibodies, aptamers can precisely recognize and bind to membrane proteins on cancer cells through their unique spatial structure with high affinity [24]. In particular, aptamers indeed do possess advantages such as high thermal/chemical stability, low immunogenicity and cheaper, easier and faster engineering, as well as rapid tissue penetration [23].

In addition, aptamers can serve in aptamer–drug conjugates (ApDCs) to precisely deliver a wide range of therapeutic agents (e.g., cytotoxic agents and others) to targeted cancer cells [25]. In this review, we primarily focus on the different strategies and the latest advances in the construction of aptamer-based drug delivery systems for targeted therapy.

## 2. CD Markers Are Great Therapeutic Targets for Hematologic Malignancy

Cell membrane proteins are currently extremely attractive targets for precision medicine in the treatment of hematologic malignancies. In a series of landmark studies, some unique surface antigens (i.e., membrane proteins) were found to be expressed much more in hematologic malignancies than in normal hematopoietic progenitor cells [26]. It means that the differences between the membrane proteins of cancer cells and normal cells are merely in expression levels [26]. Thus, membrane proteins indeed can serve as great therapeutic targets for targeted therapy [27].

**Figure 1:**
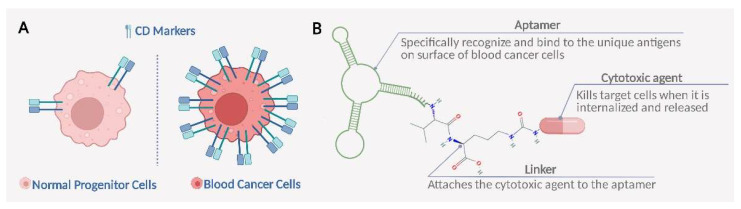
Scheme of targeted therapy for hematologic malignancies by ApDCs. (**A**) Certain CD markers are preferentially expressed in blood cancer cells, with low or no expression in normal hematologic progenitor cells. (**B**) Aptamer–drug conjugates consist of an aptamer targeting the unique membrane protein in blood cancer cells, a potent cytotoxic agent and a linker attaching the drugs to the aptamer. Created with BioRender.com.

Cluster of differentiation (CD) is a special class of membrane protein utilized for the identification of the differentiation lineage of leukemia cells [28,29]. Notably, as shown in Figure 1A, there are a number of unique CD markers more abundantly found in hematologic malignancies than in normal hematopoietic progenitor cells, indicating their potential in the development of targeted therapeutics [26]. Recently, several CD markers have been dominantly used as therapeutic target for mAbs-based immunotherapy in leukemia; therefore, we summarize the current available CD markers as potential targets for leukemia treatments (Table 1), and some CD markers targeted drugs have already been approved for clinical applications.

For example, CD33 is a single-chain, trans-membrane glycoprotein, a myeloid differentiation antigen broadly expressed on AML blast cells; therefore, it is an excellent therapeutic target for AML treatment [30,31]. In the light of this target, Mylotarg, a CD33-specific antibody–calicheamicin conjugate was first approved for CD33-positive pediatric AML treatment in 2000, while it was unfortunately withdrawn from the market in 2010 due to safety concerns such as those relating to the incidence of hepatic veno-occlusive disease, increased mortality and others [32]. Through continuous efforts to explore, Mylotarg was approved again for treatment of new indications extended to relapsed or refractory (R/R) CD33-positive AML in pediatric and older patients in 2017 [33]. On the other hand, CD20 is a B cell differentiation antigen located only in pre-B cells and mature B cells which can act as the diagnostic target in CLL and ALL [34,35,36,37,38]. Based on this target, MRG001, another ADC drug composed of chimeric anti-CD20 mAbs with anti-microtubulin agent monomethyl auristatin E (MMAE), is currently being evaluated in a phase I study in patients with CD20-positive relapsed or refractory B-cell non-Hodgkin lymphoma (NHL) [39]. 

Meanwhile, CD19 is a trans-membrane protein specifically expressed on most B cell malignancies; therefore, it can serve as an attractive biomarker for targeted therapy [40,41]. Loncastuximab tesirine is an CD19-targeted antibody–drug conjugate used for treatment of the relapsed or refractory diffuse large B-cell lymphoma (R/R DLBCL); it has proven to be a promising treatment for R/R DLBCL which is efficacious, has durable responses and is safe in this patient population [42,43]. Furthermore, anti-CD19 and/or CD21 chimeric antigen receptor (CAR) therapies utilizing human peripheral blood T lymphocytes can effectively eradicate R/R large B-cell lymphoma (LBCL) and aggressive forms of leukemia [44,45]. 

Another B lineage differentiation antigen, CD22, was also found to be highly expressed in more than 90% patients of pre-B-cell ALL and has been utilized as a therapeutic target for the construction of antibody drugs [46,47]. Inotuzumab ozogamicin is a CD22-targeted monoclonal antibody linked with cytotoxic agent calicheamicin. It has been approved for the treatment of CD22-positive relapsed or refractory B-ALL due to its superiority in improving the progression-free survival and overall survival of B-ALL patients [48,49]. Moreover, Fry et al. reported a phase I study of CD22-targeted CAR-T therapy in relapsed or refractory B-ALL patients. The results showed that anti-CD22 CAR-T cells can mediate similar potent antineoplastic effects to anti-CD19 CAR-T cells in pre-B ALL patients, and they also exhibit great efficacy in anti-CD19 immunotherapy-resistant patients with loss of or diminished surface expression of CD19 [50,51], indicating that CD markers are extremely important targets (biomarkers) for targeted therapy to eradicate hematologic malignancies. In addition to these, there are also many other sorts of targets, such as CD44 [52], CD47 [53], CD117 [54], CD123 [55] and CD134 [56] in acute leukemia, as well as CD20 [57] in chronic leukemia, which are used as specific targets for leukemia treatment, and several clinical trials are currently ongoing to assess their safety and efficacy in various clinics. 

## 3. Aptamer-Mediated Precision Therapy for Hematologic Malignancy

In fact, ADCs have achieved success in targeted therapy of hematologic malignancies, while their productions are costly as well as time consuming, and they can induce severe immune response due to the high immunogenicity [69]. As we mentioned above, aptamers (termed as chemical antibodies) are a class of single-stranded nucleic acid (ssDNA or RNA) which can precisely recognize their corresponding target molecules through their complex spatial structure with high binding affinity and have a similar function to mAbs [21]. Aptamers are generally screened from a randomized ssDNA or RNA library by an in vitro selection method called systematic evolution of ligands by exponential enrichment (SELEX) [70]. Currently, there are a few approaches (protein-, cell- and animal-model-based SELEX, as well as protein real-structure-based automatic design of aptamers by computational method) for screening aptamers with high specificity and high binding affinity (Kd values of nM to pM) [71]. More importantly, aptamers can also be screened without any knowledge of target molecules, which also makes them more attractive and promising tools for the discovery of unknown biomarkers [23,72].

Owing to aptamers’ unique chemical and biological properties, they have been widely used in cancer diagnosis and exhibit great potential for clinical treatment (i.e., targeted therapy) [73]. More importantly, aptamers can be easily conjugated with toxic agents, including chemotherapeutic molecules and toxins, as aptamer–drug conjugates (ApDCs) for target therapy of cancers not only enhance therapy efficacy, but also reduce adverse side effects in cancer patients, similar to ADCs [72]. Here, we summarize reported ApDCs for cancer treatments in Table 2. In view of the aforementioned advantages, aptamer-mediated precision therapy is deemed to be considerably efficient in the treatment of hematologic malignancies. Here, we introduce in depth a few vital cleavable and non-cleavable linkers as well as drug incorporation methods for constructing aptamer–drug conjugates. 

### 3.1. Synthesis of Aptamer–Drug Conjugates through Chemical Linkers

Synthesis of ApDCs depends on several vital research areas including the choice of an appropriate antigen target, discovery of novel, highly potent cytotoxic drugs and conjugation technology [87,88]. Importantly, the major approach for the synthesis of ApDCs is to utilize appropriate chemical linkers as a bridge to connect the aptamers and cytotoxic payloads through covalent bonds, which are key components for ApDCs to control the release of payloads to blood cancer cells, expressing the target antigen rather than to healthy cells, as shown in Figure 1B [89]. In brief, linkers require high stability in the circulation so that the payload stays connected to the aptamers when it is distributed to the tissue. Once ApDCs are precisely internalized and transported into cellular organelles of cancer cells, the linkers release the attached cytotoxic drug through the dissociation properties. Upon release, the cytotoxic drug can interfere with various cellular mechanisms, eventually leading to cell death.

**Figure 2:**
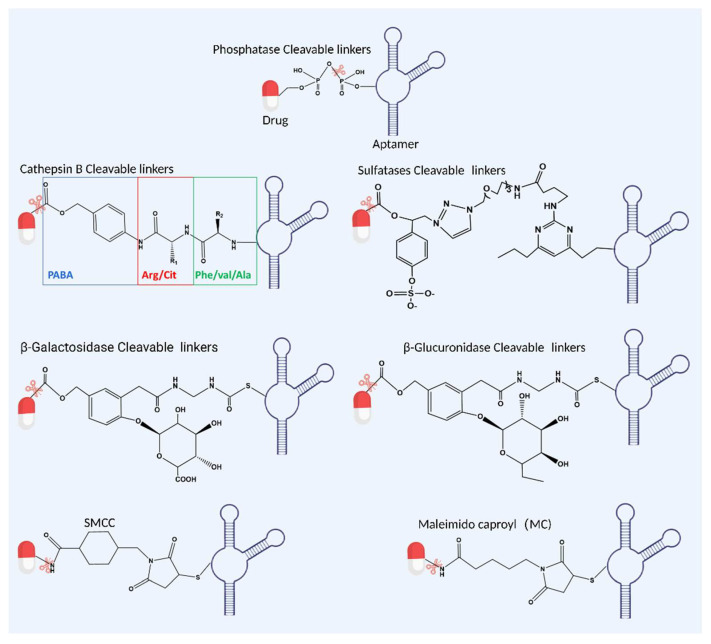
Scheme of examples of various aptamer–drug conjugates through chemical linkers. Created with BioRender.com.

Since the development of ADC drug construction, different types of linkers have been well established for the conjugation of biomacromolecules and chemical compounds. Additionally, given their dissociation properties, linkers can be divided into two categories, cleavable linkers and non-cleavable linkers. Cleavable linkers are designed to be easily cleaved enzymatically (e.g., cathepsin B, etc.) or chemically (e.g., acid-sensitive linkers and reduction-sensitive linkers), leading to the release of their payload in targeted cells [90]. Among them, cathepsin B cleavable linkers/peptide linkers are commonly used in ADCs for various payloads, including MMAE, MMAF, pyrrolobenzodiazepines (PBD) and doxorubicins (DOX) [91,92]. Currently, the valine–citrulline (Val–Cit), phenylalanine–lysine (Phe–Lys) and valine–alanine (Val–Ala) peptides are the most widely employed cathepsin B cleavable linkers due to their high stability in serum and efficient drug release toward the lysosomes of target cancer cells [93]. For instance, a Val–Cit linker with MMAE is used in brentuximab vedotin and polatuzumab vedotin for targeting CD30-positive Hodgkin lymphoma, systemic anaplastic large cell lymphoma and CD79b-positive R/R DLBCL, respectively [94,95]. Another ADC drug loncastuximab tesirine-lpyl, composed of anti-CD19 mAb conjugated with cytotoxin PBD through peptide linker Val–Ala, has been approved for the clinical treatment of large B-cell lymphoma [96,97]. Similar to cathepsin B, newly designed enzymatically cleavable linkers, such as the phosphatase cleavable linker, sulfatases cleavable linker, β-galactosidase cleavable linker and β-glucuronidases cleavable linker, have also emerged as effective linkers for drug conjugations (Figure 2).

There are a few typical chemical linkers, including cleavable and non-cleavable linkers, for connecting aptamers and anticancer drugs, for instance, cleavable linkers, such as phosphtase, cathepsin B, surfatases, β-galactosidase and β-glucuronidase cleavable linkers and non-cleavable linkers succinimidyl-4-[N-maleimidomethyl] cyclohexane-1-carboxylate (SMCC) and maleimidocaproyl (MC). 

Until now, there have been numerous chemically cleavable linkers designed to use in ADC drugs for hematologic malignancies. For example, Mylotarg, which consists of an anti-CD33 antibody and calicheamicin through an acid-cleavable hydrazone linker (i.e., chemically cleavable linker), is used for AML therapy [98]. Similarly, a hydrazone linker is also used to connect anti-CD22 mAbs to cytotoxins such as calicheamicin (inotuzumab ozogamicin) and pasudotox-tdfk (moxetumomab pasudotox-tdfk) for treatment of CD22-positive ALL and relapsed hairy cell leukemia in clinics, respectively [49,66,99,100]. 

Non-cleavable linkers maintain the coupling integrity of the aptamer and drugs throughout the entire drug action process and usually rely on complete degradation of the aptamer (or antibody) within the lysosomes to release the attached payload [90]. Mechanistically, non-cleavable linkers are unable to degrade by proteolysis and do not influence the activity of the payload after conjugation [92]. Currently, several non-cleavable alkyl and polymeric linkers are being explored in ADC development. In particular, the most representative linker is the succinimidyl-4-[N-maleimidomethyl] cyclohexane-1-carboxylate (SMCC) crosslinker, which is a heterobifunctional protein crosslinker with a sulfhydryl-reactive maleimide group and an amine-reactive N-hydroxysuccinimide (NHS) ester group [101,102] (Figure 2). It is applied in trastuzumab emtansine for the conjugation of an-HER-2 antibodies and DM1, which has been approved for the treatment of HER-2-positive breast cancer [103]. In addition, CD37-antigen-targeted naratuximab emtansine, which consists of anti-CD37 mAbs and cytotoxin DM1 through an SMCC linker, is beginning to be investigated for diffuse large B-cell lymphoma and follicular lymphoma treatment in clinical trials [104,105].

On the other hand, B-cell maturation antigen (BCMA) is found to be highly expressed on the surface of neoplastic plasma cells and plays a critical role in the proliferation, survival and tumor progression in multiple myeloma (MM). Recently, an anti-BCMA monoclonal antibody was designed to conjugate with MMAE through a non-cleavable maleimidocaproyl (MC) linker to synthesize a BCMA-targeted ADC (e.g., belantamab mafodotin-blmf) for multiple myeloma treatment [106]. 

In the light of these successes, aptamer–drug conjugates can be more easily synthesized by using these linkers and payloads due to their superior chemical properties. Zhang et al. conjugated a nucleolin target aptamer (named AS1411) with paclitaxel (PTX) through a cathepsin B–labile dipeptide linker Val–Cit [75]. As the aptamer is highly water soluble, this conjugate dramatically improved the water solubility of PTX and specifically delivered PTX into nucleolin-positive ovarian cancer cells through nucelolin-mediated micropinocytosis, resulting in notable improvement of antitumor activity and reduction of systemic toxicity. The same linker was also used for the conjugation of MMAE and MMAF with aptamers targeting EGFR or transferrin [78]. These conjugates exhibit greater anticancer activity in EGFR- and TfR-positive pancreatic cancer cells than in negative cells. Moreover, Huang et al. synthesized an aptamer–drug conjugate consisting of PTK7-targeted aptamer sgc8c linked with Dox through an acid–labile hydrazone linker [81]. This ApDC (sgc8c–Dox) effectively inhibited nonspecific uptake of Dox into non-target cells and selectively delivered Dox into targeted cancer cells. All these findings indicate that aptamers can also be conjugated with cytotoxic payload through chemical linkers to synthesize ApDCs in a similar manner to the construction of ADCs; therefore, ApDCs are promising as a supplement for ADCs in the clinical treatment of leukemia.

### 3.2. Direct Synthesis of Aptamer–Drug Conjugates

Except conjugation through chemical linkers, certain chemotherapeutic agents can be directly incorporated into aptamers to form the aptamer–drug physical conjugate due to their unique chemical properties [107,108]. Dox, a chemotherapy agent, is widely used for the treatment of a variety of malignancies such as leukemia, lymphoma, myeloma and others through intercalating into the DNA’s double helix, especially in the CG-rich region [108]. Since aptamers are able to form tertiary conformations with double-stranded regions, Dox can be physically intercalated within the CG-rich, double-stranded region of aptamers to form an aptamer–Dox conjugate [109,110]. Moreover, based on the properties of the CG-rich region, newly designed CG cargo, which contains 10~16 base pair CG repeated sequences, can be used for the linkage with aptamers as drug-intercalating sites to improve the capacity of Dox loading [111]. Yang et al. synthesized a CD33-targeted aptamer–Dox conjugate for CD33-positive AML treatment. In this study, CG-rich cargo was added into the 5′ end of aptamer S30-T1 to synthesize a S30-T1–Dox conjugate which could precisely recognize the CD33 antigen on HL-60 cells and be rapidly internalized into cells and then release the Dox, finally inducing CD33-positive AML cell death (but not CD33-negative cell death), implying that the ApDC has excellent therapeutic potential for leukemia treatment [80].

It has been reported that nucleoside analogs, such as gemcitabine and 5-fluorouracil (5-FU), are able to incorporate into the skeleton of aptamers directly due to their similar structure to that of natural nucleotides [112]. Therefore, DNA aptamers containing gemcitabine or 5-FU are considered to be chemically synthesized by using solid-phase DNA synthesis techniques [113]. Wang et al. reported that five copies of 5-FU-linked phosphoramidite can be site-specifically loaded onto the aptamer by automated, solid-phase DNA synthesis, which has proven to be highly effective for delivering 5-FU into targeted cancer cells, indicating that such conjugates can also have therapeutic potential in clinical applications for leukemia treatment [82]. Additionally, gemcitabine is also able to incorporate into RNA aptamers through transcription reactions catalyzed by special RNA polymerase, such as a mutant T7 RNA polymerase (Y639F), which efficiently utilizes non-canonical NTP for synthesizing RNAs [79,113]. Likewise, Ray et al. successfully synthesized an EGFR-targeted aptamer–gemcitabine polymer (Gem–E07 polymer) through an enzymatic reaction by taking advantage of mutant T7 RNA polymerase in which seven cytosine sites of aptamer E07 are actually enzymatically replaced by gemcitabine monophosphates. Moreover, the Gem–E07 conjugate also showed a strong inhibition effect on growth of EGFR-positive pancreatic cancer cells after internalization through clathrin-mediated endocytosis [79]. Taken together, ApDCs can be rapidly synthesized with a nucleoside analog and chemically modified with diverse functional groups at either the 5′ or the 3′ end to facilitate site-specific conjugation, as well as increase the drug loading capacity.

## 4. Aptamer–T Cell (AP–T) Targeted Therapy for Hematologic Malignancy

Engineering immune T cells for cancer treatment is a rapidly emerging area in cell-based immunotherapy [114,115]. The most remarkable success is the use of CD19 and/or CD21 chimeric antigen receptor T (CAR-T) cells for treating hematologic malignancies. In clinic, CAR-T cell therapies have shown superior antitumor efficacy in patients with refractory B cell malignancies, including ALL and non-Hodgkin lymphoma [116,117]. However, due to the integration of DNA into the host cell genome by retroviral elements, as well as severe cytokine storm symptoms and potential carcinogenicity in patients, the clinical use of CAR-T cells for cancer immunotherapy is largely restricted [118]. Thus, development of a non-protein antigen receptor and non-viral new T cell therapy is indeed required.

Since the aptamer has similar properties to mAbs, it is supposed to replace the antigen receptor on the surface of CAR-T cells for targeting cancer cells [119]. Notably, unnatural sugars can be rationally designed to enable preferential metabolic labeling of cancer cells and protein for the development of tumor-targeted therapy [120,121]. Liu et al., for the first time, generated aptamer–CD3^+^ T cells by using N-azidomannosamine (ManNAz) sugar metabolic labeling and click chemistry (i.e., a non-viral method) against cancer [120]. In brief, they initially conjugated azide onto the cellular surface of human CD3^+^ T cells through glycol-metabolic labeling, and a dibenzocyclooctyne (DBCO)-labeled DNA aptamer could conjugate with azide through a bio-orthogonal copper-free click reaction, finally generating aptamer–CD3^+^ T cells. As anticipated, synthesized aptamer–T cells specifically bound to tumor cells and exhibited stronger antitumor effects with less cytotoxicity as well as non-carcinogenicity in vitro and in vivo, suggesting that aptamer–T cell therapy can be used as a potential new immunotherapy strategy for the treatment of hematologic malignancies. 

Unlike the aptamer–T cell therapy, bi-specific aptamers that consist of two aptamers (as bivalent or multivalent structures) can concurrently bind to two different targets on the same cells or different cells [122]. Bi-specific aptamers are designed to specifically target two different antigens, one is multidrug-resistance-associated membrane protein 1 (MRP1), which is highly expressed in chemotherapy-resistant tumor cells, while another is CD28 on T lymphocytes, which functions to provide the co-stimulatory signals required for T cell activation and survival [123]. The engineered, bi-specific, therapeutic, chimeric aptamers (MRP1-CD28) could activate the tumor-infiltrating lymphocyte (TILs) against melanoma tumors and showed strong antitumor activity through inducing an immune response in vivo [114,123,124]. Similar work was also performed by using bi-specific aptamers to form junctional T cell and cancer cell complexes [125], and T cells were further activated in situ by CD3/CD28 T cell activator beads. Such aptamer–guided T cell immunotherapy showed strong antitumor immunity against multiple tumor models with high therapeutic efficacy. Therefore, activation of the immune system against cancer by bi-specific aptamers provides a smart approach through which personalized cancer therapy seems to be plausible.

## 5. Aptamer–PROTAC Conjugates (ApPCs)

It is worth noting that certain subtypes of leukemia are caused by the formation of abnormal oncogenic proteins (e.g., PML-RARα, BCR-ABL, BET) [126,127], while the emergence of several small molecules, such as arsenic trioxide (As_2_O_3_) and imatinib, successfully cures such types of leukemia. However, drug resistance resulting from mutations in oncoproteins leads to treatment failure in some relapsed patients. Moreover, there are also numerous leukemia oncoproteins that cannot be handled by kinase inhibitors or small molecule degraders [128,129,130]. 

Fortunately, an important advance that is likely to have a major impact on targeting such targets is the advent of proteolysis-targeting chimeras (PROTACs) [131]. PROTACs are bivalent and bi-functional small molecules that facilitate degradation of oncogenic protein through the ubiquitin–proteasome system (UPS) [132]. Mechanistically, PROTACs contain an E3 ligase-recruiting ligand, a linker and a target-protein-binding ligand [132,133]. Currently, a number of PROTACs are being developed for the degradation of leukemia oncogenic proteins such as BCR-ABL, CDK, BTK, BET and FLT3 [134]. However, conventional PROTACs are limited due to poor cell membrane permeability and lack of tumor specificity. He et al. recently developed a novel aptamer–PROTAC conjugate to improve the specificity of PROTACs [135]. Here, a BET-targeted PROTAC-PRO was conjugated with a nucleolin-targeted aptamer (named AS) through a cleavable ester–disulfide linker. This designed aptamer–PROTAC conjugate (named APR) could be selectively internalized into nucleolin-overexpressed tumors cells through receptor-mediated endocytosis, and PRO molecules were intracellularly released after the ester and disulfide bond was broken. Moreover, APR improved tumor targeting ability and BET degradation, leading to increased antitumor activity as well as decreased toxicity in vitro and in vivo, indicating that aptamer–PROTAC conjugates are an effective approach for enhancing the clinical value of PROTAC drugs. 

On the other hand, for some undruggable transcription factors such as Ras and Myc, there is no small molecule available for specific binding due to their intrinsic structural disorder and lack of small molecule binding pockets [128,129,130]. Additionally, owing to remarkable specificity and binding affinity, aptamers can also be utilized as target molecules for the construction of PROTAC that is able to degrade these oncoproteins [136]. Zhang et al. designed a conjugate of nucleolin-targeted aptamer AS1411 and a small molecule ligand of E3 ligase VHL via a DBCO–azide click reaction [137]. This PROTAC molecule ZL216 is able to promote the formation of a nucleolin–ZL216–VHL ternary complex, resulting in potent nucleolin degradation in breast cancer cells as well as in xenograft models. Collectively, aptamer-based PROTAC seems to be a reasonable approach for the treatment of undruggable transcription-factor-driven hematologic malignancies, through precisely degrading their oncogenic proteins, and the curing of the diseases.

## 6. Conclusions and Perspective

In this review, we comprehensively discussed aptamers in clinic application for hematologic malignancies therapy. Especially, we described in depth the aptamer–drug conjugates (ApDCs) and the importance of chemical linkers (non-cleavable and cleavable) for connecting aptamers and cytotoxin agents to synthesize ApDCs. Actually, ApDCs are efficient means of delivering therapeutic cytotoxin to targeted blood cancer cells through recognition of their targets and release of their cytotoxin payloads to kill cancer cells. Compared with ADCs, aptamers can be conjugated with drugs through diverse approaches (i.e., linker-based and non-linker-based approaches), implying that ApDCs have a broader range of choice of payload. Furthermore, aptamers are easier to chemically modify, and their production cost is much lower than ADCs, indicating that ApDCs have greater commercial prospects. More importantly, aptamers have lower immunogenicity and do not induce severe immunoreaction in vivo. Although certain aptamers are beginning to be investigated for clinical use and be assessed for their safety and efficacy, the barriers to the translation of aptamers into the clinic are still challenges. For instance, due to the low serum stability and fast renal excretion of aptamers, proper chemical modifications are necessary to improve their weakness. In addition, novel aptamer screening platforms are urgently needed to be developed for the high-throughput selection of aptamers with high binding affinity and specificity. On the other hand, other novel therapeutic approaches, such as aptamer–T cell therapy and aptamer–PROTAC conjugates, are also very exciting new ideas, and personalized hematologic malignancies therapy seems to be plausible through these strategies in the near future.

## Figures and Tables

**Table 1:** CD markers as therapeutic target for leukemia treatment.

Classification		Biomarker	Description	Agent	Ref.
		CD33	Belongs to Siglecs family; in approximately 85% to 90% AML patients.	Gemtuzumab ozogamicin CAR-T (phase 1)	[32]
		CD44	Strongly expressed on all AML cells.	RO5429083 with cytarabine (phase 1), CAR-T (phase 1/2)	[52,58]
		CD47	Overexpressed in leukemic blasts and progenitors, a macrophage immune checkpoint, protects cells from phagocytosis.	Lemzoparlima (phase 1/2a), magrolimab (5F9) with azacitidine (phase 1b)	[53,59,60]
	Acute Myeloid Leukemia (AML)	CD117	Also named C-kit, a tyrosine kinase receptor, expressed in more than 90% of AML patients with physiological HSPC and leukemic blasts.	MGTA-117 (phase 1)	[54,61]
Acute Leukemia		CD123	Mainly expressed on AML leukemic stem cells.	CSL362 (phase 1), flotetuzumab (phase 1) CAR-T (phase 2)	[32,55]
		CD134	Also named OX40, belongs to NGFR/TNFR superfamily, mainly expressed on Teffs and Tregs. OX40–OX40L interaction promotes NK cells in AML.	n.a.	[56]
		CD170	Also named siglec-5, upregulated during granulocyte maturation, overexpressed on the AML non-M3 phenotypes.	n.a.	[62]
	Acute Lymphocytic Leukemia (ALL)	CD19	80% of ALL expressed moderate to high levels of CD19.	Blinatumomab	[63,64]
		CD22	Highly expressed on leukemic cells from most R/R B-ALL patients.	Inotuzumab ozogamicin (phase 2), moxetumomab pasudotox-tdfk	[65,66]
Chronic Leukemia	Chronic Lymphocytic Leukemia (CLL)	CD20	Expressed in B-cell-derived tumor cells, such as CLL.	Ofatumumab (phase 2), obinutuzumab (phase 2)	[57,67,68]

**Table 2:** Aptamer–drug conjugates for cancer treatment.

Aptamer	Target	Drug	Cancer	Reference
AS1411	Nucleolin	Dox	Liver Cancer	[74]
		Pacitaxel	Ovarian Cancer	[75]
		Gemcitabine	Pancreatic Cancer	[76]
P19	PANC-1 cell	MMAE	Pancreatic Cancer	[77]
		DM1	Pancreatic Cancer	[77]
E07	EGFR	MMAE	Pancreatic Cancer	[78]
		MMAF	Pancreatic Cancer	[78]
		Gemcitabine	Pancreatic Cancer	[79]
Waz	Transferrin	MMAE	Pancreatic Cancer	[78]
		MMAF	Pancreatic Cancer	[78]
S30-T1	CD33	Dox	Acute Myeloid Leukemia	[80]
Sgc8	PTK7	Dox	Acute Lymphoblastic Leukemia	[81]
		5-FU	Colorectal Cancer	[82]
EpDT3	EpCAM	Dox	Colorectal Cancer	[83]
AP-1	CD133	Dox	Anaplastic Thyroid Cancer	[84]
HB-5	HER-2	Dox	Breast Cancer	[85]
MA-3	MUC-1	Dox	Lung CancerBreast Cancer	[86]

## Data Availability

No new data were created or analyzed in this study. Data sharing is not applicable to this article.

## References

[1] Hodson D.J., Screen M., Turner M. (2019). RNA-binding proteins in hematopoiesis and hematological malignancy. Blood.

[2] Sheth A., de Melo V.A., Szydlo R., Szydlo R., Macdonald D.H., Reid A.G., Wagner S.D. (2009). Specific patterns of chromosomal gains and losses associate with t(3;14), t(8;14), and t(14;18) in diffuse large B-cell lymphoma. Cancer Genet. Cytogenet..

[3] Ozery-Flato M., Linhart C., Trakhtenbrot L., Izraeli S., Shamir R. (2011). Large-scale analysis of chromosomal aberrations in cancer karyotypes reveals two distinct paths to aneuploidy. Genome Biol..

[4] Rustad E.H., Yellapantula V.D., Glodzik D., Maclachlan K.H., Diamond B., Boyle E.M., Ashby C., Blaney P., Gundem G., Hultcrantz M. (2020). Revealing the Impact of Structural Variants in Multiple Myeloma. Blood Cancer Discov..

[5] Bergh J.C. (1990). Gene amplification in human lung cancer. The myc family genes and other proto-oncogenes and growth factor genes. Am. Rev. Respir. Dis..

[6] Young D.J., Nguyen B., Li L., Higashimoto T., Levis M.J., Liu J.O., Small D. (2021). A Method for Overcoming Plasma Protein Inhibition of Tyrosine Kinase Inhibitors. Blood Cancer Discov..

[7] Soverini S., Martelli M., Bavaro L., Benedittis C.D., Iurlo A., Galimberti S., Pregno P., Bonifacio M., Lunghi F., Castagnetti F. (2019). Detection of Actionable BCR-ABL1 Kinase Domain (KD) Mutations in Chronic Myeloid Leukemia (CML) Patients with Failure and Warning Response to Tyrosine Kinase Inhibitors (TKIs): Potential Impact of Next-Generation Sequencing (NGS) and Droplet Digital PCR (ddPCR) on Clinical Decision Making. Blood.

[8] Shastri A., Gonzalez-Lugo J., Verma A. (2020). Understanding FLT3 Inhibitor Resistance to Rationalize Combinatorial AML Therapies. Blood Cancer Discov..

[9] Zhang X.H., Chen J., Han M.Z., Huang H., Jiang E.L., Jiang M., Lai Y.R., Liu D.H., Liu Q.F., Liu T. (2021). The consensus from The Chinese Society of Hematology on indications, conditioning regimens and donor selection for allogeneic hematopoietic stem cell transplantation: 2021 update. J. Hematol. Oncol..

[10] Walker B.A. (2020). The Chromosome 13 Conundrum in Multiple Myeloma. Blood Cancer Discov..

[11] Zarbo A., Axelson A., Lim H.W., Kohen L.L., Schneider S.F., Yeager D. (2020). Common Cutaneous Side Effects of Anti-cancer Agents. Practical Guide to Dermatology: The Henry Ford Manual.

[12] Stoddart A., Wang J., Fernald A.A., Davis E.M., Johnson C.R., Hu C., Cheng J.X., McNerney M.E., Le Beau M.M. (2020). Cytotoxic Therapy-Induced Effects on Both Hematopoietic and Marrow Stromal Cells Promotes Therapy-Related Myeloid Neoplasms. Blood Cancer Discov..

[13] Padma V.V. (2015). An overview of targeted cancer therapy. Biomedicine (Taipei).

[14] Zhang T., Yang J., Vaikari V.P., Beckford J.S., Wu S., Akhtari M., Alachkar H. (2020). Apolipoprotein C2—CD36 Promotes Leukemia Growth and Presents a Targetable Axis in Acute Myeloid Leukemia. Blood Cancer Discov..

[15] Panchagnula R., Dey C.S. (1997). Monoclonal antibodies in drug targeting. J. Clin. Pharm. Ther..

[16] Hafeez U., Parakh S., Gan H.K., Scott A.M. (2020). Antibody-Drug Conjugates for Cancer Therapy. Molecules.

[17] Kantarjian H., Short N.J., DiNardo C., Stein E.M., Daver N., Perl A.E., Wang E.S., Wei A., Tallman M. (2021). Harnessing the benefits of available targeted therapies in acute myeloid leukaemia. Lancet Haematol..

[18] Pittaluga S., Nicolae A., Wright G.W., Melani C., Roschewski M., Steinberg S., Huang D., Staudt L.M., Jaffe E.S. (2020). Wilson, W.H. Gene Expression Profiling of Mediastinal Gray Zone Lymphoma and Its Relationship to Primary Mediastinal B-cell Lymphoma and Classical Hodgkin Lymphoma. Blood Cancer Discov..

[19] Estey E.H. (2020). Acute myeloid leukemia: 2021 update on risk-stratification and management. Am. J. Hematol..

[20] Schurch C.M. (2018). Therapeutic Antibodies for Myeloid Neoplasms-Current Developments and Future Directions. Front. Oncol..

[21] Chesi M., Stein C.K., Garbitt V.M., Sharik M.E., Asmann Y.W., Bergsagel M., Riggs D.L., Welsh S.J., Meermeier E.W., Kumar S.K. (2020). Monosomic loss of MIR15A/MIR16-1 is a driver of multiple myeloma proliferation and disease progression. Blood Cancer Discov..

[22] Maimaitiyiming Y., Ye L., Yang T., Yu W., Naranmandura H. (2022). Linear and Circular Long Non-Coding RNAs in Acute Lymphoblastic Leukemia: From Pathogenesis to Classification and Treatment. Int. J. Mol. Sci..

[23] Shigdar S., Ward A.C., De A., Yang C.J., Wei M., Duan W. (2011). Clinical applications of aptamers and nucleic acid therapeutics in haematological malignancies. Br. J. Haematol..

[24] Sicco E., Baez J., Fernández M., Fernández M., Cabral P., Moreno M., Cerecetto H., Calzada V. (2020). Sgc8-c Aptamer as a Potential Theranostic Agent for Hemato-Oncological Malignancies. Cancer Biother. Radiopharm..

[25] Kim D.H., Seo J.M., Shin K.J., Yang S.G. (2021). Design and clinical developments of aptamer-drug conjugates for targeted cancer therapy. Biomater Res..

[26] Eladl E., Tremblay-LeMay R., Rastgoo N., Musani R., Chen W., Liu A., Chang H. (2020). Role of CD47 in Hematological Malignancies. J. Hematol. Oncol..

[27] Louvet C., Nadeem O., Smith E.L. (2021). Finding the optimal partner to pair with bispecific antibody therapy for multiple myeloma. Blood Cancer Discov..

[28] Rezaeeyan H., Shahrabi S., McKee T.D., Saki N. (2018). The expression of CD markers in solid tumors: Significance in metastasis and prognostic value. Histol. Histopathol..

[29] Russ A., Hua A.B., Montfort W.R., Rahman B., Riaz I.B., Khalid M.U., Carew J.S., Nawrocki S.T., Persky D., Anwer F. (2018). Blocking “don’t eat me” signal of CD47-SIRPalpha in hematological malignancies, an in-depth review. Blood Rev..

[30] Walter R.B. (2014). The role of CD33 as therapeutic target in acute myeloid leukemia. Expert Opin. Ther. Targets.

[31] Dillon L.W., Ghannam J., Nosiri C., Gui G., Goswami M., Calvo K.R., Lindblad K.E., Oetjen K.A., Wilkerson M.D., Soltis A.R. (2021). Personalized Single-Cell Proteogenomics to Distinguish Acute Myeloid Leukemia from Nonmalignant Clonal Hematopoiesis. Blood Cancer Discov..

[32] Rubnitz J.E., Kaspers G.J.L. (2021). How I treat pediatric acute myeloid leukemia. Blood.

[33] Norsworthy K.J., Ko C.-W., Lee J.E., Liu J., John C.S., Przepiorka D., Farrell A.T., Pazdur R. (2018). FDA Approval Summary: Mylotarg for Treatment of Patients with Relapsed or Refractory CD33-Positive Acute Myeloid Leukemia. Oncologist.

[34] Abou-El-Enein M., Elsallab M., Feldman S.A., Fesnak A.D., Heslop H.E., Marks P., Till B.G., Bauer G., Savoldo B. (2021). Scalable Manufacturing of CAR T cells for Cancer Immunotherapy. Blood Cancer Discov..

[35] Raman T., Mohanraj S., Muthu A., Prabhakar V., Ramakrishnan B., Vaidhyanathan L., Easow J., Raja T. (2021). Independent diagnostic utility of CD20, CD200, CD43 and CD45 in chronic lymphocytic leukaemia. Leuk. Lymphoma.

[36] Alduailej H., Kanfar S., Bakhit K., Raslan H., Alsaber A., Bashawri L., Aldayel A., Alanezi K. (2020). Outcome of CD20-positive Adult B-cell Acute Lymphoblastic Leukemia and the Impact of Rituximab Therapy. Clin. Lymphoma Myeloma Leuk..

[37] Tsuzuki S., Yasuda T., Kojima S., Kawazu M., Akahane K., Inukai T., Imaizumi M., Morishita T., Miyamura K., Ueno T. (2020). Targeting MEF2D-fusion Oncogenic Transcriptional Circuitries in B-cell Precursor Acute Lymphoblastic Leukemia. Blood Cancer Discov..

[38] Kläsener K., Jellusova J., Andrieux G., Salzer U., Böhler C., Steiner S.N., Albinus J.B., Cavallari M., Süß B., Voll R.E. (2021). CD20 as a gatekeeper of the resting state of human B cells. Proc. Natl. Acad. Sci. USA.

[39] Song Y., Guo Y., Wang Z., Wu M., Peng W., Sun L., Sun J.H., Li M., Zhu J. (2021). A Dose Escalation Phase Ia Study of Anti-CD20 Antibody Drug Conjugate, MRG001 in Relapsed/Refractory Advanced Non-Hodgkin Lymphom. Blood.

[40] Katz B.Z., Herishanu Y. (2014). Therapeutic targeting of CD19 in hematological malignancies: Past, present, future and beyond. Leuk Lymphoma.

[41] Haloupek N. (2020). The Landscape of Blood Cancer Research Today-and Where the Field Is Headed. Blood Cancer Discov..

[42] Simon S., Riddell S.R. (2020). Dual Targeting with CAR T Cells to Limit Antigen Escape in Multiple Myeloma. Blood Cancer Discov..

[43] Caimi P.F., Ai W., Alderuccio J.P., Ardeshna K.M., Hamadani M., Hess B., Kahl B.S., Radford J., Solh M., Stathis A. (2021). Loncastuximab tesirine in relapsed or refractory diffuse large B-cell lymphoma (LOTIS-2): A multicentre, open-label, single-arm, phase 2 trial. Lancet Oncol..

[44] Abramson J.S. (2020). Anti-CD19 CAR T-Cell Therapy for B-Cell Non-Hodgkin Lymphoma. Transfus. Med. Rev..

[45] Maciocia N.C., Burley A., Nannini F., Wawrzyniecka P.A., Neves M.P., Karpanasamy T., Ferrari M., Marafioti T., Onuoha S.C., Khwaja A.I. (2021). Anti-CD21 Chimeric Antigen Receptor (CAR)-T Cells for T Cell Acute Lymphoblastic Leukaemia (T-ALL). Blood.

[46] Lanza F., Maffini E., Rondoni M., Massari E., Faini A.C., Malavasi F. (2020). CD22 Expression in B-Cell Acute Lymphoblastic Leukemia: Biological Significance and Implications for Inotuzumab Therapy in Adults. Cancers.

[47] Shaffer A.L., Phelan J.D., Wang J.Q., Huang D., Wright G.W., Kasbekar M., Choi J., Young R.M., Webster D.E., Yang Y. (2021). Overcoming Acquired Epigenetic Resistance to BTK Inhibitors. Blood Cancer Discov..

[48] Kantarjian H., Thomas D., Jorgensen J., Kebriaei P., Jabbour E., Rytting M., York S., Ravandi F., Garris R., Kwari M. (2013). Results of inotuzumab ozogamicin, a CD22 monoclonal antibody, in refractory and relapsed acute lymphocytic leukemia. Cancer.

[49] Thota S., Advani A. (2017). Inotuzumab ozogamicin in relapsed B-cell acute lymphoblastic leukemia. Eur. J. Haematol..

[50] Fry T.J., Shah N.N., Orentas R.J., Stetler-Stevenson M., Yuan C.M., Ramakrishna S., Wolters P., Martin S., Delbrook C., Yates B. (2018). CD22-targeted CAR T cells induce remission in B-ALL that is naive or resistant to CD19-targeted CAR immunotherapy. Nat. Med..

[51] Zhu H., Deng H., Mu J., Lyu C., Jiang Y., Deng Q. (2021). Anti-CD22 CAR-T Cell Therapy as a Salvage Treatment in B Cell Malignancies Refractory or Relapsed After Anti-CD19 CAR-T therapy. Onco Targets Ther..

[52] Yu X., Munoz-Sagredo L., Streule K., Muschong P., Bayer E., Walter R.J., Gutjahr J.C., Greil R., Concha M.L., Müller-Tidow C. (2021). CD44 loss of function sensitizes AML cells to the BCL-2 inhibitor venetoclax by decreasing CXCL12-driven survival cues. Blood.

[53] Qi J., Li J., Jiang B., Jiang B., Liu H., Cao X., Zhang M., Meng Y., MA X., Jia Y. (2020). A Phase I/IIa Study of Lemzoparlimab, a Monoclonal Antibody Targeting CD47, in Patients with Relapsed and/or Refractory Acute Myeloid Leukemia (AML) and Myelodysplastic Syndrome (MDS): Initial Phase I Results. Blood.

[54] Rodriguez C., Jin T., Jawde R.A., Saber W., Baz R., His E., Kalaycio M., Sobecks R., Sekeres M., Advani A. (2006). c-Kit (CD117) Expression Is a Poor Prognostic Factor for Relapse and Overall Survival in Patients with Newly Diagnosed AML. Blood.

[55] Smith B.D., Roboz G.J., Walter R.B., Altman J.K., Ferguson A., Curcio T.J., Orlowski K.F., Garrett L., Busfield S.J., Barnden M. (2014). First-in Man, Phase 1 Study of CSL362 (Anti-IL3Rα / Anti-CD123 Monoclonal Antibody) in Patients with CD123+ Acute Myeloid Leukemia (AML) in CR at High Risk for Early Relapse. Blood.

[56] Schumacher C.E., Nuebling T., Hofmann M., Schmiedel B.J., Kanz L., Jung G., Salih H.R. (2012). The Role of OX40 and Its Ligand in Acute Myeloid Leukemia: Expression, Function and Modulation of NK Cell Anti-Leukemia Reactivity. Blood.

[57] Byrd J.C. (2019). Targeting CD20 takes the backseat in CLL. Blood.

[58] Al-Madhoun N.Y., Gadhoum S.Z., Merzaban J.S. (2012). ERK1/2 Pathway Is Required for Differentiation of AML Triggered by Anti-CD44 Monoclonal Antibodies. Blood.

[59] Sallman D.A., Asch A.S., Malki M.M.A., Lee D.J., Donnellan W.B., Marcucci G., Kambhampati S., Daver N.G., Garcia-Manero G., Komrokji R.S. (2019). The First-in-Class Anti-CD47 Antibody Magrolimab (5F9) in Combination with Azacitidine Is Effective in MDS and AML Patients: Ongoing Phase 1b Results. Blood.

[60] Melo Garcia L., Barabé F. (2021). Harnessing Macrophages through the Blockage of CD47, Implications for Acute Myeloid Leukemia. Cancers.

[61] Myburgh R., Kiefer J.D., Russkamp N.F., Magnani C.F., Nuñez N., Simonis A., Pfister S., Wilk C.M., McHugh D., Friemel J. (2020). Anti-human CD117 CAR T-cells efficiently eliminate healthy and malignant CD117-expressing hematopoietic cells. Leukemia.

[62] Yang M., Jiang G., Li W., Qiu K., Zhang M., Carter C.M., Al-Quran S.Z., Li Y. (2014). Developing aptamer probes for acute myelogenous leukemia detection and surface protein biomarker discovery. J. Hematol. Oncol..

[63] Papayannidis C., Paolini S., Santoro A., Robustelli V., Soverini S., Benedittis C.D., Imbrogno E., Terragna C., Rorà A.G., Parisi S. (2016). Blinatumomab is safe and effective in relapsed and MRD-positive B-ALL CD19+ patients: The Bologna Compassionate Program Experience. Blood.

[64] Lee D.W., Stetler-Stevenson M., Yuan C.M., Shah N.N., Delbrook C.P., Yates B., Zhang H., Zhang L., Kochenderfer J.N., Rosenberg S.A. (2016). Long-Term Outcomes Following CD19 CAR T Cell Therapy for B-ALL Are Superior in Patients Receiving a Fludarabine/Cyclophosphamide Preparative Regimen and Post-CAR Hematopoietic Stem Cell Transplantation. Blood.

[65] Chevallier P., Leguay T., Doubek M., Huguet F., Šálek C., Cabannes A., Wartiovaara-Kautto U., Saillard C., Raffoux E., Cluzeau T. (2021). Fractionated Inotuzumab Ozogamicin Combined with Low-Intensity Chemotherapy Provides Very Good Outcome in Older Patients with Newly Diagnosed CD22+ Philadelphia Chromosome-Negative B-Cell Precursor Acute Lymphoblastic Leukemia: First Results from the EWALL-INO Study. Blood.

[66] Kreitman R.J., Dearden C.E., Zinzani P.L.L., Delgado J., Robak T., le Coutre P., Gjertsen B.T., Troussard X., Roboz G.J., Karlin L. (2019). Moxetumomab Pasudotox-Tdfk in Heavily Pretreated Patients with Relapsed/Refractory Hairy Cell Leukemia (HCL): Long-Term Follow-up from the Pivotal Phase 3 Trial. Blood.

[67] Wierda W.G., Jewell R.C., Kipps T.J., Dürig J., Griškevičius L., Stilgenbauer S., Smolej L., Hess G., Hernandez-Ilizaliturri F.J., Padmanabhan S. (2011). Correlations between Ofatumumab Exposure and Treatment Outcomes for Patients with Chronic Lymphocytic Leukemia (CLL) Treated with Frontline Ofatumumab, Fludarabine, and Cyclophosphamide Chemoimmunotherapy. Blood.

[68] Brown J.R., O′Brien S., Kingsley C.D., Eradat H.A., Pagel J.M., Hirata J., McIver T., Morariu-Zamfir R., Kipps T.J. (2019). Durable remissions with obinutuzumab-based chemoimmunotherapy: Long-term follow-up of the phase 1b GALTON trial in CLL. Blood.

[69] Ni S., Zhuo Z., Pan Y., Yu Y., Li F., Liu J., Wang L., Wu X., Li D., Wan Y. (2021). Recent Progress in Aptamer Discoveries and Modifications for Therapeutic Applications. ACS Appl. Mater. Interfaces.

[70] Darmostuk M., Rimpelova S., Gbelcova H., Ruml T. (2015). Current approaches in SELEX: An update to aptamer selection technology. Biotechnol. Adv..

[71] Zhou J., Rossi J. (2017). Aptamers as targeted therapeutics: Current potential and challenges. Nat. Rev. Drug Discov..

[72] Xuan W., Peng Y., Deng Z., Peng T., Kuai H., Li Y., He J., Jin C., Liu Y., Wang R. (2018). A basic insight into aptamer-drug conjugates (ApDCs). Biomaterials.

[73] Wu X., Chen J., Wu M., Zhao J.X. (2015). Aptamers: Active targeting ligands for cancer diagnosis and therapy. Theranostics.

[74] Trinh T.L., Zhu G., Xiao X., Puszyk W.M., Sefah K., Wu Q., Tan W., Liu C. (2015). A Synthetic Aptamer-Drug Adduct for Targeted Liver Cancer Therapy. PLoS ONE.

[75] Zhang J., Chen R., Chen F., Chen M., Wang Y. (2015). Nucleolin targeting AS1411 aptamer modified pH-sensitive micelles: A dual-functional strategy for paclitaxel delivery. J. Control. Release.

[76] Park J.Y., Cho Y.L., Chae J.R., Moon S.H., Cho W.G., Choi Y., Lee S.J., Kang W.J. (2018). Gemcitabine-Incorporated G-Quadruplex Aptamer for Targeted Drug Delivery into Pancreas Cancer. Mol. Ther. Nucleic Acids.

[77] Yoon S., Huang K.W., Reebye V., Spalding D., Przytycka T.M., Wang Y., Swiderski P.M., Li L., Armstrong B., Reccia I. (2017). Aptamer-Drug Conjugates of Active Metabolites of Nucleoside Analogs and Cytotoxic Agents Inhibit Pancreatic Tumor Cell Growth. Mol. Ther. Nucleic Acids.

[78] Kratschmer C., Levy M. (2018). Targeted Delivery of Auristatin-Modified Toxins to Pancreatic Cancer Using Aptamers. Mol. Ther. Nucleic Acids.

[79] Ray P., Cheek M.A., Sharaf M.L., Li N., Ellington A.D., Sullenger B.A., Shaw B.R., White R.R. (2012). Aptamer-mediated delivery of chemotherapy to pancreatic cancer cells. Nucleic Acid Ther..

[80] Yang C., Wang Y., Ge M.H., Fu Y., Hao R., Islam K., Huang P., Chen F., Sun J., Hong D. (2019). Rapid identification of specific DNA aptamers precisely targeting CD33 positive leukemia cells through a paired cell-based approach. Biomater. Sci..

[81] Huang Y.F., Shangguan D., Liu H., Phillips J.A., Zhang X., Chen Y., Tan W. (2009). Molecular assembly of an aptamer-drug conjugate for targeted drug delivery to tumor cells. Chembiochem.

[82] Wang R., Zhu G., Mei L., Xie Y., Ma H., Ye M., Qing F., Tan W. (2014). Automated modular synthesis of aptamer-drug conjugates for targeted drug delivery. J. Am. Chem. Soc..

[83] Subramanian N., Raghunathan V., Kanwar J.R., Kanwar R.K., Elchuri S.V., Khetan V., Krishnakumar S. (2012). Target-specific delivery of doxorubicin to retinoblastoma using epithelial cell adhesion molecule aptamer. Mol. Vis..

[84] Ge M.H., Zhu X.H., Shao Y.M., Wang C., Huang P., Wang Y., Jiang Y., Maimaitiyiming Y., Chen E., Yang C. (2021). Synthesis and characterization of CD133 targeted aptamer-drug conjugates for precision therapy of anaplastic thyroid cancer. Biomater. Sci..

[85] Liu Z., Duan J., Song Y., Ma J., Wang F., Lu X., Yang X. (2012). Novel HER2 aptamer selectively delivers cytotoxic drug to HER2-positive breast cancer cells In Vitro. J. Transl. Med..

[86] Hu Y., Duan J., Zhan Q., Wang F., Lu X., Yang X. (2012). Novel MUC1 aptamer selectively delivers cytotoxic agent to cancer cells In Vitro. PLoS ONE.

[87] Zhu G., Niu G., Chen X. (2015). Aptamer-Drug Conjugates. Bioconjugate Chem..

[88] Bruno J.G. (2013). A review of therapeutic aptamer conjugates with emphasis on new approaches. Pharmaceuticals.

[89] Qi J., Zeng Z., Chen Z., Nipper C., Liu X., Wan Q., Chen J., Tung C., Zu Y. (2022). Aptamer-Gemcitabine Conjugates with Enzymatically Cleavable Linker for Targeted Delivery and Intracellular Drug Release in Cancer Cells. Pharmaceuticals.

[90] Bargh J.D., Isidro-Llobet A., Parker J.S., Spring D.R. (2019). Cleavable linkers in antibody–drug conjugates. Chem. Soc. Rev..

[91] Sheyi R., de la Torre B.G., Albericio F. (2022). Linkers: An Assurance for Controlled Delivery of Antibody-Drug Conjugate. Pharmaceutics.

[92] McCombs J.R., Owen S.C. (2015). Antibody drug conjugates: Design and selection of linker, payload and conjugation chemistry. AAPS J..

[93] Kostova V., Désos P., Starck J.B., Kotschy A. (2021). The Chemistry Behind ADCs. Pharmaceuticals.

[94] van de Donk N.W., Dhimolea E. (2012). Brentuximab vedotin. Mabs-Austin.

[95] Poreba M. (2020). Protease-activated prodrugs: Strategies, challenges, and future directions. FEBS J..

[96] Hamadani M., Radford J., Carlo-Stella C., Caimi P.F., Reid E.G., O’Connor O.A., Feingold J.M., Ardeshna K., Townsend W.M., Solh M.M. (2021). Final results of a phase 1 study of loncastuximab tesirine in relapsed/refractory B-cell non-Hodgkin lymphoma. Blood.

[97] Su Z., Xiao D., Xie F., Liu L., Wang Y., Fan S., Zhou X., Li S. (2021). Antibody–drug conjugates: Recent advances in linker chemistry. Acta Pharm. Sin. B.

[98] Vollmar B.S., Frantz C., Schutten M.M., Zhong F., del Rosario G., Go M., Yu S., Leipold D.D., Kamath A.V., Ng C. (2021). Calicheamicin Antibody-Drug Conjugates with Improved Properties. Mol. Cancer Ther..

[99] Cordo′ V., van der Zwet J.C.G., Canté-Barrett K., Pieters R., Meijerink J. (2020). T-cell Acute Lymphoblastic Leukemia: A Roadmap to Targeted Therapies. Blood Cancer Discov..

[100] Kaushal A., Nooka A.K., Carr A.R., Pendleton K.E., Barwick B.G., Manalo J., McCachren S.S., Gupta V.A., Joseph N.S., Hofmeister C.C. (2021). Aberrant Extrafollicular B Cells, Immune Dysfunction, Myeloid Inflammation, and MyD88-Mutant Progenitors Precede Waldenstrom Macroglobulinemia. Blood Cancer Discov..

[101] Lyon R.P., Setter J.R., Bovee T.D., Doronina S.O., Hunter J.H., Anderson M.E., Balasubramanian C.L., Duniho S.M., Leiske C.I., Li F. (2014). Self-hydrolyzing maleimides improve the stability and pharmacological properties of antibody-drug conjugates. Nat. Biotechnol..

[102] Chen Q., Gabathuler R. (2004). Efficient Synthesis of Doxorubicin Melanotransferrin p97 Conjugates Through SMCC Linker. Synth. Commun..

[103] Barok M., Joensuu H., Isola J. (2014). Trastuzumab emtansine: Mechanisms of action and drug resistance. Breast Cancer Res..

[104] Palomba M.L., Younes A. (2013). In the spotlight: A novel CD37 antibody-drug conjugate. Blood.

[105] Liu Z., Filip I., Gomez K., Engelbrecht D., Meer S., Lalloo P.N., Patel P., Perner Y., Zhao J., Wang J. (2020). Genomic characterization of HIV-associated plasmablastic lymphoma identifies pervasive mutations in the JAK-STAT pathway. Blood Cancer Discov..

[106] Markham A. (2020). Belantamab Mafodotin: First Approval. Drugs.

[107] Li X., Zhao Q., Qiu L. (2013). Smart ligand: Aptamer-mediated targeted delivery of chemotherapeutic drugs and siRNA for cancer therapy. J. Control. Release.

[108] Bagalkot V., Farokhzad O.C., Langer R., Jon S. (2006). An aptamer-doxorubicin physical conjugate as a novel targeted drug-delivery platform. Angew. Chem. Int. Ed. Engl..

[109] Xiang D., Shigdar S., Qiao G., Wang T., Kouzani A.Z., Zhou S.F., Kong L., Li Y., Pu C., Duan W. (2015). Nucleic acid aptamer-guided cancer therapeutics and diagnostics: The next generation of cancer medicine. Theranostics.

[110] Macdonald J., Denoyer D., Henri J., Jamieson A., Burvenich I., Pouliot N., Shigdar S. (2020). Bifunctional Aptamer-Doxorubicin Conjugate Crosses the Blood-Brain Barrier and Selectively Delivers Its Payload to EpCAM-Positive Tumor Cells. Nucleic Acid Ther..

[111] Yang C., Jiang Y., Hao S.H., Yan X.Y., Hong D.F., Naranmandura H. (2021). Aptamers: An emerging navigation tool of therapeutic agents for targeted cancer therapy. J. Mater. Chem. B.

[112] Zhu L., Yang J., Ma Y., Zhu X., Zhang C. (2022). Aptamers Entirely Built from Therapeutic Nucleoside Analogues for Targeted Cancer Therapy. J. Am. Chem. Soc..

[113] Sousa R., Padilla R. (1995). A mutant T7 RNA polymerase as a DNA polymerase. EMBO J..

[114] Fernández de Larrea C., Staehr M., Lopez A.V., Ng K.Y., Chen Y., Godfrey W.D., Purdon T.J., Ponomarev V., Wendel H.G., Brentjens R.J. (2020). Defining an Optimal Dual-Targeted CAR T-cell Therapy Approach Simultaneously Targeting BCMA and GPRC5D to Prevent BCMA Escape-Driven Relapse in Multiple Myeloma. Blood Cancer Discov..

[115] Silvestri G., Trotta R., Stramucci L., Ellis J.J., Harb J.G., Neviani P., Wang S., Eisfeld A.K., Walker C.J., Zhang B. (2020). Persistence of Drug-Resistant Leukemic Stem Cells and Impaired NK Cell Immunity in CML Patients Depend on MIR300 Antiproliferative and PP2A-Activating Functions. Blood Cancer Discov..

[116] Lai X., Gu X., Tsao S.T., Zhang Q.J., Liu Y., Yaxian J., Yan L., Chun J., Zhang R., Du Y. (2017). Double CD19/CD22 Chimeric Antigen Receptor-Modified T Cells for the Treatment of Stage IV Relapsed and Refractory Follicular Lymphoma. Blood.

[117] van de Donk N.W.C.J., Themeli M., Usmani S.Z. (2021). Determinants of Response and Mechanisms of Resistance of CAR T-cell Therapy in Multiple Myeloma. Blood Cancer Discov..

[118] June C.H., O’Connor R.S., Kawalekar O.U., Ghassemi S., Milone M.C. (2018). CAR T cell immunotherapy for human cancer. Science.

[119] Kacherovsky N., Cardle I.I., Cheng E.L., Yu J.L., Baldwin M., Salipante S.J., Jensen M.C., Pun S.H. (2019). Traceless aptamer-mediated isolation of CD8(+) T cells for chimeric antigen receptor T-cell therapy. Nat. Biomed. Eng..

[120] Liu C.G., Wang Y., Liu P., Yao Q., Zhou Y., Li C., Zhao Q., Liu G., Zhang X. (2020). Aptamer-T Cell Targeted Therapy for Tumor Treatment Using Sugar Metabolism and Click Chemistry. ACS Chem. Biol..

[121] Wang H., Mooney D.J. (2020). Metabolic glycan labelling for cancer-targeted therapy. Nat. Chem..

[122] Liu X., Yan H., Liu Y., Chang Y. (2011). Targeted cell-cell interactions by DNA nanoscaffold-templated multivalent bispecific aptamers. Small (Weinh. Der Bergstr. Ger.).

[123] Soldevilla M.M., Villanueva H., Casares N., Lasarte J.J., Bendandi M., Inogés S., de Cerio A.L., Pastor F. (2016). MRP1-CD28 bi-specific oligonucleotide aptamers: Target costimulation to drug-resistant melanoma cancer stem cells. Oncotarget.

[124] Lancman G., Sastow D.L., Cho H.J., Jagannath S., Madduri D., Parekh S., Richard S., Richter J., Sanchez L., Chari A. (2021). Bispecific Antibodies in Multiple Myeloma: Present and Future. Blood Cancer Discov..

[125] Yang Y., Sun X., Xu J., Cui C., Safari Yazd H., Pan X., Zhu Y., Chen X., Li X., Li J. (2020). Circular Bispecific Aptamer-Mediated Artificial Intercellular Recognition for Targeted T Cell Immunotherapy. ACS Nano.

[126] Maimaitiyiming Y., Wang Q.Q., Yang C., Ogra Y., Lou Y., Smith C.A., Hussain L., Shao Y.M., Lin J., Liu J. (2021). Hyperthermia Selectively Destabilizes Oncogenic Fusion Proteins. Blood Cancer Discov..

[127] Rosselló-Tortella M., Ferrer G., Esteller M. (2020). Epitranscriptomics in Hematopoiesis and Hematologic Malignancies. Blood Cancer Discov..

[128] Romine K.A., Nechiporuk T., Bottomly D., Jeng S., McWeeney S.K., Kaempf A.J., Corces M.R., Majeti R., Tyner J.W. (2021). Monocytic Differentiation and AHR Signaling as Primary Nodes of BET Inhibitor Response in Acute Myeloid Leukemia. Blood Cancer Discov..

[129] Moore A.R., Rosenberg S.C., McCormick F., Malek S. (2020). RAS-targeted therapies: Is the undruggable drugged?. Nat. Rev. Drug Discov..

[130] Schmidt C.R., Achille N.J., Kuntimaddi A., Boulton A.M., Leach B.I., Zhang S., Zeleznik-Le N.J., Bushweller J.H. (2020). BCOR Binding to MLL-AF9 Is Essential for Leukemia via Altered EYA1, SIX, and MYC Activity. Blood Cancer Discov..

[131] Ogawa S. (2020). Deciphering the Clonal Origin of Relapsed Acute Lymphoblastic Leukemia in Children. Blood Cancer Discov..

[132] Khan S., He Y., Zhang X., Yuan Y., Pu S., Kong Q., Zheng G., Zhou D. (2020). PROteolysis TArgeting Chimeras (PROTACs) as emerging anticancer therapeutics. Oncogene.

[133] Edmondson S.D., Yang B., Fallan C. (2019). Proteolysis targeting chimeras (PROTACs) in ‘beyond rule-of-five’ chemical space: Recent progress and future challenges. Bioorganic Med. Chem. Lett..

[134] Bricelj A., Steinebach C., Kuchta R., Gütschow M., Sosič I. (2021). E3 Ligase Ligands in Successful PROTACs: An Overview of Syntheses and Linker Attachment Points. Front. Chem..

[135] He Y., Khan S., Huo Z., Lv D., Zhang X., Liu X., Yuan Y., Hromas R., Xu M., Zheng G. (2020). Proteolysis targeting chimeras (PROTACs) are emerging therapeutics for hematologic malignancies. J. Hematol. Oncol..

[136] He S., Gao F., Ma J., Ma H., Dong G., Sheng C. (2021). Aptamer-PROTAC Conjugates (APCs) for Tumor-Specific Targeting in Breast Cancer. Angew. Chem. Int. Ed. Engl..

[137] Zhang L., Li L., Wang X., Liu H., Zhang Y., Xie T., Zhang H., Li X., Peng T., Sun X. (2022). Development of a Novel PROTAC using the Nucleic Acid Aptamer as a Targeting Ligand for Tumor Selective Degradation of Nucleolin. Mol. Ther.-Nucleic Acids.

